# Parenting quality and early childhood development: evidence from different rural subpopulations in China

**DOI:** 10.1186/s40359-025-03580-5

**Published:** 2025-11-18

**Authors:** Lei Wang, Lijuan Zheng, Yu Bai, Sarah-Eve Dill, Scott Rozelle

**Affiliations:** 1 International Business School, Shaanxi Normal University, Xi’an, China; 2 Teaching Quality Monitoring and Evaluation Center, Fuzhou University, Fuzhou, China; 3 School of Economics, China Institute for Vitalizing Border Areas and Enriching the People, Minzu University of China, Beijing, China; 4 Stanford Center on China’s Economy and Institutions, Stanford University, Stanford, United States of America

**Keywords:** Parenting quality, Early childhood development, Rural subpopulation, China

## Abstract

**Background:**

The quality of parenting can affect the developmental outcomes of young children. This study aims to investigate the associations between parenting quality and the early childhood development of children under age 3 across four major rural subpopulations in China.

**Methods:**

Using a stratified cluster sampling method, 760 children aged 6–36 months and their primary caregivers in four rural subpopulations from four provinces and a metropolis in China were surveyed. Child development was assessed by the Third Edition of the Bayley Scales of Infant and Toddler Development. Parenting quality was measured using the Family Care Indicators. Data were analyzed using descriptive statistics, t-tests, multivariable regression analysis, and linear regression analysis.

**Results:**

Across the four subpopulations, prevalences of delays of the sample children in four domains — cognition, language, social-emotional, and motor development are 52%, 45%, 52%, and 19%, respectively. The proportion of children with any type of delay is 82%, while over half (53%) have delays in at least two areas, and 27% have delays in three or more areas. Child’s mother as the primary caregiver, maternal education levels, and family asset values are all positively associated with the quality of parenting. Notably, low levels of parenting quality in rural China are linked to high rates of developmental delays.

**Conclusions:**

This study demonstrates that the level of parenting quality is significantly associated with early childhood developmental outcomes. Results highlight the need for raising investments in family care to improve early childhood development in different rural subpopulations in China.

**Supplementary Information:**

The online version contains supplementary material available at 10.1186/s40359-025-03580-5.

## Introduction

The first three years of life are a critical period of rapid brain growth and the development of cognitive and non-cognitive abilities [1–3]. For this reason, early childhood development (ECD) outcomes (e.g., cognitive, language, social-emotional, and motor abilities) lay the foundation for long-term skill development and human capital accumulation [4, 5]. Numerous studies have linked developmental outcomes in early childhood to later education attainment, employment outcomes, and earnings [6–8].

Because early brain development appears to be influenced by the environment and psychosocial stimulation, the quality of parenting is often considered one of the possible factors that contribute to a child’s ECD [9, 10]. A stimulating home learning environment, parental involvement in the development of their children through stimulating activities such as reading, singing, and storytelling, as well as exposing young children to diverse experiences may reflect higher quality of parenting [11]. Stimulation in the home or the production of a quality home environment are thought to be helpful of improving the development of children [12]. These actions and conditions of family care have been associated with more favorable cognitive and non-cognitive developmental outcomes [13].

The literature has used measures of family care and the home environment to assess the quality of stimulation and learning opportunities for children. The most widely used set of validated measurements for the quality of parenting in developing countries is the Family Care Indicators (FCIs). FCIs provide a means to assess the social and physical conditions that are considered to influence the cognitive and non-cognitive development of children. Studies in various developing countries have focused on specific aspects of family care measured by the FCIs, such as the sources and variety of play materials, engagement of caregivers in activities with children, and the presence of adult books and magazines in the home [12, 14]. These studies have found that the aspects are closely associated with child developmental outcomes, with children who receive higher-quality parenting as measured by the FCIs showing significantly better cognitive and non-cognitive development [9, 15, 16].

Across rural China, studies have found high rates of developmental delays among children under age 3. A study of 1,442 rural children aged 24–36 months in Shaanxi Province found that the rate of cognitive delay was nearly 50% [17]. In a systematic review study, the rates of cognitive, language, and social-emotional delays of young rural children from 14 provinces of China were found to be 45%, 46%, and 36%, respectively [18]. The rates of developmental delays in rural China are much higher than those of the urban population, in which only about 15% of the children have delays [19].

Research on the causes of these delays in rural China has pointed to a lack of interactive parenting practices as one possible source of poor developmental outcomes among rural children [18, 20–22]. No studies, however, have examined the multiple aspects of FCIs that comprise family care (sources and variety of play materials, interactive play activities between caregivers and children, the presence of adult books, and the presence of magazines and newspapers) and their associations to child developmental outcomes in rural China.

There also have been few efforts to document the relationship between parenting quality and ECD across different subpopulations of developing settings. International studies of parenting quality have tended to focus on only one rural or peri-urban population [16, 23]. Similarly, previous research on parenting quality in rural China has focused on either the mountainous regions of rural China [20, 24] or one narrow geographic range [21, 25]. Residents in rural China live in a variety of populations, however, in addition to the remote and mountainous rural villages on which previous research has focused. For example, many rural communities in central China have higher population densities and income than those in remote mountainous villages [26]. A growing number of rural residents have also moved to resettlement communities, which are residential areas with housing subsidized by the state to consolidate scattered populations that originally lived in mountainous villages [27]. In addition, many rural families migrated to China’s large urban centers, often seeking better education opportunities for their children, and settled in migrant enclaves [28]. To our knowledge, no study in China or internationally has attempted to examine the associations between parenting quality and developmental delays across different rural subpopulations (More information on the four rural subpopulations in China can be found in the Supplementary materials).

Given vastly different individual experiences and family environments across rural subpopulations, we expect children in these subpopulations to show variations in developmental outcomes. The goal of this study is to provide an understanding of the associations between FCIs and ECD across four major rural subpopulations in China: mountainous rural, central plains, resettlement, and migrant. To this end, we have four objectives. First, we report the findings of a large-scale survey of child developmental outcomes and compare developmental outcomes across the four subpopulations. Second, we report and compare indicators of family care across the four subpopulations. Third, we identify which child, caregiver, and family characteristics are associated with higher quality of parenting. Finally, we examine the associations between FCIs and ECD outcomes, including cognitive, language, social-emotional, and motor development.

## Methods

### Study design and sample

A cross-sectional study was designed to examine the associations between parenting quality and ECD across four major rural subpopulations in China, from Shaanxi, Hebei, Yunnan, Henan, and Beijing. We used a multistage clustering strategy to select the sample and 760 caregiver-child dyads were included in this study. The data used in this study were collected in five areas of China: (A) a northwestern province (Shaanxi), (B) a northeastern province (Hebei), (C) a southwestern province (Yunnan), (D) a central province (Henan), and (E) a metropolis in northern China (Beijing). Based on the division of China’s rural populations found in the Communique on Major Data of the Second National Agricultural Census of China [29], we classified our total sample from these five areas into four rural subpopulations: mountainous rural, central plains, resettlement, and migrant. The mountainous rural populations in our sample are from mountain areas of two western provinces, which are among the poorest regions of China.

We conducted sample size calculations, which indicated that at least 124 caregiver-child dyads were required to detect the expected correlation (with 80% power using a two-sided 5% significance level), accounting for 10 control covariates. Based on this calculation, our research plan required random sampling of households until the final sample exceeded a minimum requirement of 496 caregiver-child dyads.

Sample selection followed a multi-stage clustering strategy. Overall, 760 children aged 6–36 months and their families were included. Specifically, 157 were recruited from mountainous rural populations in two provinces (Hebei and Yunnan), 157 from central plains populations, 163 from resettlement populations in Shaanxi and Henan provinces, and 245 migrant populations from three urban areas: Xi’an City 1 (in Shaanxi), Zhengzhou City (in Henan), and Beijing. A summary of the distribution and location of the households in each of these subsamples can be found in Table 1.

**Table 1:** Distribution of rural subpopulations

Location of study	Year	Population type	Age of children	Number of observations
Provinces Hebei and Yunnan	2016	Mountainous Rural	6–36 months	195
Provinces Shaanxi and Henan	2017	Central Plains	6–36 months	157
Provinces Shaanxi and Henan	2017	Resettlement	6–36 months	163
Xi’an City (in Shaanxi); Zhengzhou City (in Henan); Beijing	2017	Migrant	6–36 months	245

Throughout this paper, we used sampling weights in order to more accurately represent the share of these subpopulations in China’s rural population overall. The proportions for each subpopulation in rural China are 37.7% for mountainous rural populations, 1.4% for resettlement populations, 42.0% for central rural populations, and 18.8% for migrant populations. We calculated the sampling weights, using the following formula: sampling weight = proportion of subpopulation in total population/proportion of subpopulation in each sample. The subpopulation proportions in the sample are as follows: 86.0% for mountainous rural populations, 4.0% for resettlement populations, 3.8% for central rural populations, and 6.1% for migrant populations. Using these formulas, the sampling weight for mountainous rural populations is 01.45 (which is equivalent to 37.3%/26%); for resettlement populations the sampling weight is 0.07 (1.4%/21%); for central rural populations the sampling weight is 2.0 (42%/21%); and for migrant populations the sampling weight is 0.59 (18.8%/32%).

## Measures

### Early childhood development

To assess ECD, we used the Third Edition of the Bayley Scales of Infant and Toddler Development (Bayley-III), a comprehensive scale and the internationally recognized golden standard for assessing ECD outcomes of children aged 6–42 months. The Bayley-III has been formally adapted to the Chinese language and has been widely used across rural China [26, 30, 31]. The results of the Bayley-III are categorized into five standardized scales, four of which we used in the present study: cognitive, language, social-emotional, and motor. The cognitive scale (91 items) assesses information processing, counting, and number skills; the language scale (97 items) assesses both receptive and expressive communication skills; the motor scale (138 items) assesses fine and gross motor skills; and the social-emotional scale (175 items) measures functional emotional skills, including internal emotional regulation and social responsiveness [32].

The cognitive, language, and motor scales were administered one-on-one to each child by trained enumerators using a standardized set of toys and a detailed scoring sheet. The enumerators evaluated the child based on his or her performance on a number of tasks, for example, “calms down when being picked up” (cognitive scale), “regards persons momentarily” (language scale), “hands are fisted” (motor scale). The social-emotional scale was implemented by asking the child’s primary caregiver a series of questions to assess the child’s mastery of functional emotional skills, including self-regulation and interest in the world, communication needs, interacting and building relationships with others, using emotions in an interactive and purposeful manner, and using emotional signals or gestures to solve problems.

Each of the four subscales accounts for the child’s gestational age and chronological age when calculating the final score. Upon completion of the test, raw Bayley-III scores were converted to composite scores according to the Bayley-III guidelines [33]. According to the guidelines, we first obtained raw scores for each domain from the standardized tasks administered by the study’s enumerators. Second, the raw scores were converted into age-normed scaled scores provided by the Manual of the Bayley-III. Third, the scaled scores from relevant subtests (e.g., Receptive and Expressive Communication for the language domain) were summed and converted into composite scores using standardized conversion tables (also provided by the Manual of the Bayley-III). A higher composite score indicates better child development. For our analysis, we standardized the raw scores of the cognitive, language, social-emotional, and motor development domains, using age-conditional means and standard deviations estimated by non-parametric regressions. Specifically, we first estimated age-conditional means and standard deviations using non-parametric standardization, and then the estimated statistics were used to compute age-adjusted internal z-scores. This non-parametric standardization method is less sensitive to outliers and a small sample size within age-category and yields normally distributed standardized scores with a mean of zero across all of the study’s age ranges by month [13]. A higher score indicates better development. In the current study, Cronbach’s alpha of the scale was 0.69, indicating good reliability.

### Parenting quality

To assess the parenting quality, we administered the FCIs survey to the primary caregivers of all sample children. The United Nations Children’s Fund (UNICEF) developed the FCI questionnaire to measure parenting quality in developing countries [34]. Survey items are divided into five dimensions: sources of play materials, variety of play materials, play activities, household books, and magazines or newspapers. All items were scored as a binary, based on the presence or absence of play material or activity (yes = 1, no = 0). The primary caregivers were also asked to report the number of *household books* for adults as well as the number of *magazines or newspapers* in the household. In this paper, we define the variable - *household books* - as a dummy variable which takes a value of 1 if the household reported having at least 2 adult books, and 0 otherwise. The variable - *magazines or newspaper*s - takes a value of 1 if the household was reported having at least one magazine or newspaper, and 0 otherwise. Studies that use large samples of children have found FCIs to be a reliable survey-based indicator of the quality of family care and predictive of child developmental outcomes, particularly in poor rural areas of developing countries [12]. In the current study, the scale of FCIs demonstrated good internal consistency. In this study, Cronbach’s alpha for FCIs was 0.73.

### Child and household characteristics

A primary caregiver-reported questionnaire was used to collect data on child and household characteristics. The characteristics of the child include the child’s gender, age in months, and whether the child was born prematurely. The characteristics of the household include the relationship of primary caregiver to the child (e.g., mother or other), the mother’s education level, the mother’s age, parental occupations, whether the household receives Minimum Living Standard Guarantee payments (a government assistance program for the lowest-income families nationwide, henceforth referred to as welfare payments), and family asset value. To determine family asset value, the polychoric principal component analysis was used to construct a family asset index based on whether the household had the following items: tap water, toilet, water heater, washing machine, computer, internet, refrigerator, air conditioner, motorized or electric bicycle, and car. The analysis used polychoric principal component analysis to estimate the underlying correlations matrix between the above set of binary asset ownership indicators. The first principal component was extracted and used as the family asset index, which can explain the largest share of common variance across these household assets. This method has been widely used in previous studies conducted in rural China [17, 19–22]. According to the recommendation of Xie [35], parental occupations were classified into five categories: farming, working at home, self-supporting industry or commerce, government employees, and full-time caregivers.

Based on previous studies in rural China [14, 17–22], we included variables that measured child and household characteristics that were collected in the primary caregiver-reported questionnaire as covariates in our analysis, as these covariates have been regarded as fundamental factors that influence early childhood developmental outcomes and caregiver parenting in the context of rural China.

### Procedure

We recruited university students majoring in education and early childhood to serve as enumerators (43 enumerators in total). Prior to the survey, all enumerators underwent an intensive training course led by a certified and experienced team in the use of the Bayley-III (the certification was given by Jingmei company, which is the only institution that has a patent of the Chinese version of the Bayley-III). The training lasted one week and included theoretical instruction, demonstrations, hands-on practice sessions, and 2.5 days supervised pilot assessment in the field. Enumerators administered the test in the home of each child. The caregiver was required to stay with the child but was not allowed to assist the child during the administration of the tests. To ensure the quality and reliability of the assessment, we implemented quality control measures, including daily field supervision by team leaders and video recordings of the assessment that were available for review by the survey team leaders. During the survey, enumerators were required to attend meetings each night to discuss the challenges that they encountered during the day and receive ongoing feedback from senior researchers that had rich experiences in Bayley-III test and data collection. For the assessment of parenting quality, the enumerators administered the FCIs survey by interviewing the primary caregiver of each child. The primary caregiver (typically either the child’s mother or grandmother) was identified in each family as the individual who is the most responsible for the child’s care.

### Statistical analysis

All statistical analyses were conducted using STATA 18.0 Version. All statistical tests are two-sided. Our statistical analysis comprises four parts. First, we measure and compare the prevalence of developmental delays. A developmental delay on any scale of the Bayley-III is defined as a score of one or more SDs below the mean of the norm population [32]. Previous studies have shown that scores of the Bayley-III may overestimate the development levels of children and so the delay cut-offs need to be higher than the original cut-off scores in order to have results from studies using Bayles-III scales consistent with studies using other Bayles scales [36–39]. In our analysis, we used the cut-offs for each of the domains set by these studies. Specifically, the mean (standard deviation, or SD) of healthy children is 105 (9.6) for the cognitive scale [36], 109 (12.3) for the language scale [37], 100 (15) for the social-emotional scale [38], and 107 (14) for the motor scale [36, 39]. These cut-offs have been widely used in studies conducted in rural China [18, 20, 22]. However, readers need to interpret these results with caution, given that the selected cut-offs have not been formally validated in the local context. For comparison, we also define a severe developmental delay as a score of two or more SDs below the mean of the norm population.

Second, we compare the prevalence of developmental delays and FCI scores across the four rural subpopulations. To do so, we conduct two separate sets of *t*-tests to compare the differences between sample observations in rural mountainous populations and each of the three other subpopulations. One set of t-tests examines differences in the developmental delays of sample children, while the other focuses on differences in FCI. We use mountainous rural populations as the reference group as the most existing research on ECD in rural China has been conducted in poor mountainous regions.

Third, we adopt multivariable regression analyses to examine the associations between child and household characteristics and the composite FCI score. To estimate these correlations, we construct a regression model as follows:

$$\:{FCI}_{i}=\alpha\:+\beta\:{X}_{i}+{\epsilon\:}_{i}$$

where the dependent variable, $\:{FCI}_{i}\:$indicates the composite FCI score for the family of child i, and $\:{X}_{i}$ refers to the child and household characteristics of child i. Child characteristics include child’s gender and age, and whether the child was born prematurely. Household characteristics include whether the mother is the primary caregiver, maternal age, maternal educational level, parental occupations, whether the household receives Minimum Living Standard Guarantee (MLSG), and household asset index. We accounted for clustering at the village level. Because there may be intragroup correlations among the samples who live in the same village or same community, cluster-adjusted standard errors are used account for within-cluster correlation. We also included dummy variables for rural subpopulations and county fixed effects to control for unobserved, time-invariant differences across subpopulations and counties.

Finally, we measure the associations between FCIs and ECD outcomes. To do so, we adopt an Ordinary Least Squares (OLS) regression modeling approach and construct a model as follows:

$$\:{Developmental\:Outcomes}_i={\beta\:}_0+{\beta\:}_1{FCI}_i+{\beta\:}_2X_i+{\epsilon\:}_i$$

where the dependent variable, $\:{Developmental\:Outcomes}_i$, indicates the standardized Bayley-III test scores (cognitive, language, motor, and social-emotional scale scores) of infant i. All Bayley-III scores are continuous variables. The variable $\:{FCI}_{i}\:$represents the composite FCI score and scores for each of the five dimensions of the FCI scales (source of play materials, variety of play materials, play activities, number of household books, and number of magazines and newspapers) for the family of infant *i*. $\:{X}_{i}$ is a vector of covariates that are included to capture child and household characteristics. We account for clustering at the village level. We also include dummy variables for rural subpopulations and county fixed effects to control for unobserved, time-invariant differences across subpopulations and counties.

## Results

### Child and household characteristics

Table 2 shows the socioeconomic and demographic characteristics of study participants across the four subpopulations in our sample. Of the 760 children in the full sample, the average age is 21 months. Slightly over half (54%) of the sample children are male, and 5% were born prematurely. For 64% of the children in the sample, their mother is their primary caregiver, while grandmothers are the primary caregivers for another 25% of the sample children; The primary caregivers for the remaining 11% of the sample children are other family members like the father, grandfather, uncle, or aunt. Of the mothers in our sample, 36% have completed more than 12 years of schooling, and 75% are over 25 years of age. Of the sample families, 11% receive welfare payments.`a`

**Table 2:** Summary statistics

	Full sample	Mountainous Rural populations	Central rural populations	Resettlement populations	Migrant populations	Difference between populations		
	Mean	Mean	Mean	Mean	Mean	*p*-value		
	(SD)	(SD)	(SD)	(SD)	(SD)			
						(2)-(3)	(2)-(4)	(2)-(5)
	(1)	(2)	(3)	(4)	(5)	(6)	(7)	(8)
Child characteristics								
Age (in months)	21.88	24.90	20.42	19.88	19.28	0.000	0.000	0.000
	(7.85)	(3.59)	(9.03)	(9.19)	(9.14)			
Male (1 = yes)	0.54	0.56	0.55	0.53	0.48	0.993	0.954	0.352
	(0.50)	(0.50)	(0.50)	(0.50)	(0.50)			
Premature (1 = yes)	0.05	0.04	0.05	0.05	0.09	0.983	0.99	0.236
	(0.23)	(0.20)	(0.22)	(0.22)	(0.28)			
Household characteristics								
Primary caregiver	0.64	0.68	0.57	0.55	0.71	0.170	0.067	0.965
(1 = mother)	(0.48)	(0.47)	(0.50)	(0.50)	(0.46)			
Maternal age	0.75	0.79	0.71	0.74	0.78	0.304	0.723	0.987
(1 = above 25 years)	(0.43)	(0.40)	(0.46)	(0.44)	(0.42)			
Maternal education level	0.36	0.28	0.31	0.33	0.60	0.995	0.891	0.000
(1 = 12 years or higher)	(0.48)	(0.46)	(0.46)	(0.47)	(0.49)			
Household receives MLSG	0.11	0.11	0.12	0.09	0.09	0.996	0.886	0.942
(1 = yes)	(0.32)	(0.32)	(0.33)	(0.28)	(0.29)			
Household asset index	−0.23	−0.76	−0.15	−0.32	0.65	0.000	0.007	0.000
	(1.27)	(1.30)	(1.14)	(1.32)	(0.89)			
Observations	760	195	157	163	245			

*MLSG* Minimum Living Standard Guarantee

Across the four different subpopulations, we find that mothers in migrant populations have the highest educational attainment. Of the mothers from migrant populations, 60% have attained at least 12 years of education compared to 28% of mothers in mountainous rural populations, 31% in central plains populations, and 33% in resettlement populations. Migrant populations also have the highest household asset value, followed by central plains populations and resettlement populations. Mountainous rural populations have the lowest household asset value in our sample. Of all the subpopulations, migrant populations and resettlement populations have the lowest shares of sampled residents who receive welfare payments (both 9%).

### Early childhood developmental outcomes across subpopulations

The ECD outcomes of the full sample and each subpopulation sample are shown in Table 3. In the full sample, the average standardized scores for cognitive, language, social-emotional, and motor development are 98.29, 98.48, 89.36, and 105.37, respectively. Comparisons across rural subpopulations reveal substantial disparities in developmental outcomes. Children from mountainous rural areas perform worst across most domains. Their mean cognitive score (95) is significantly lower than those in the central plains (99.27), resettlement communities (99.26), and migrant populations (102.53). Similarly, their average social-emotional score (88.15) is significantly lower than that of migrant children (93.04). Migrant children also score higher in the language (102.07) and motor domains (106.93) compared to children in mountainous areas (97.37 and 106.87, respectively), with the language difference reaching statistical significance (*p* =.007).

**Table 3:** Child developmental scores

	Full sample	Mountainous Rural populations	Central rural populations	Resettlement populations	Migrant populations	Difference between populations		
	Mean	Mean	Mean	Mean	Mean	(P-value)		
	(SD)	(SD)	(SD)	(SD)	(SD)			
						(2)-(3)	(2)-(4)	(2)-(5)
	(1)	(2)	(3)	(4)	(5)	(6)	(7)	(8)
Cognitive score	98.29	95.00	99.27	99.26	102.53	0.038	0.036	0.000
	(13.95)	(10.85)	(15.25)	(15.01)	(14.92)			
Language score	98.48	97.37	97.94	95.53	102.07	0.986	0.674	0.007
	(14.09)	(10.98)	(14.56)	(13.56)	(17.63)			
Social-emotional score	89.36	88.15	88.92	84.79	93.04	0.970	0.183	0.006
	(14.49)	(13.25)	(15.05)	(11.80)	(15.27)			
Motor score	105.37	106.87	103.45	101.23	106.93	0.266	0.012	1.000
	(16.08)	(14.80)	(17.05)	(16.69)	(15.97)			

Table 4. represents the prevalence of developmental delays among children. The results show that 52% of the children experience cognitive developmental delay, 45% experience language developmental delay, 52% experience social-emotional developmental delay, and 19% experience motor developmental delay. The proportion of children with any type of delay is 81%, while over half (52%) experience delays in at least two domains and 26% in three or more areas. When comparing developmental delays across subpopulations, we find that cognitive delays are significantly lower in central plains populations (46%), resettlement populations (49%), and migrant populations (40%) when compared to mountainous populations (65%). Migrant populations also show significantly lower rates of language delays (36%) and social-emotional delays (41%) compared to mountainous populations (where the rates of language and social-emotional delays are 49% and 55%, respectively). There is no significant difference in language delays in central plains populations or resettlement populations when they are compared to mountainous populations. In addition, children in migrant populations have a significantly lower probability of experiencing any one, two, or three types of delay (73%, 42%, and 15%, respectively) when compared to those in mountainous populations (86%, 59%, and 29%, respectively). Central plains and resettlement populations have significantly higher rates of motor delay (25% and 29%, respectively) compared to mountainous populations (13%), although these rates of motor delay are much closer to those of a healthy population than are the rates of delay for cognitive, language, and social-emotional development in our sample.

**Table 4.** Child developmental delays

	**Full sample**	**Mountainous Rural populations**	**Central rural populations**	**Resettlement populations**	**Migrant populations**	**Difference between populations**		
	Frequency	Frequency	Frequency	Frequency	Frequency	(P-value)		
	(Percentages)	(Percentages)	(Percentages)	(Percentages)	(Percentages)			
						(2)-(3)	(2)-(4)	(2)-(5)
	(1)	(2)	(3)	(4)	(5)	(6)	(7)	(8)
Cognitive delay	376	64	73	80	97	0.008	0.031	0.000
(1=yes)	(52.03)	(64.61)	(46.50)	(49.07)	(39.59)			
Language delay	338	95	73	83	87	0.982	0.982	0.054
(1=yes)	(45.29)	(48.72)	(46.50)	(50.92)	(35.51)			
Social-emotional delay	398	107	86	104	101	1.000	0.414	0.043
(1=yes)	(52.35)	(54.87)	(54.78)	(63.80)	(54.78)			
Motor delay	151	25	40	47	39	0.027	0.002	0.875
(1=yes)	(18.93)	(12.82)	(25.48)	(28.83)	(15.92)			
Any of delayed	616	168	129	141	178	0.815	1.000	0.004
(1=yes)	(81.91)	(86.15)	(82.17	(86.50)	(72.65)			
Any of two types of delay	395	116	81	96	102	0.532	1.000	0.003
(1=yes)	(52.76)	(59.49)	(51.59)	(58.90)	(41.63)			
Any of three types of delay	197	56	48	57	36	0.985	0.618	0.012
(1=yes)	(26.90)	(28.72)	(30.57)	(34.97)	(14.69)			
Four types of delay	55	13	14	20	8	0.879	0.235	0.589
(1=yes)	(7.04)	(6.67)	(8.90)	(12.27)	(8.92)			
Severe cognitive delay	126	39	26	29	32	0.868	0.959	0.300
(1=yes)	(17.20)	(20.00)	(16.56)	(17.79)	(13.06)			
Severe language delay	112	23	25	29	35	0.748	0.452	0.748
(1=yes)	(14.08)	(11.79)	(15.92)	(17.79)	(14.29)			
Severe social-emotional delay	74	18	14	26	16	1.000	0.170	0.802
(1=yes)	(8.68)	(9.23)	(8.92)	(15.95)	(6.53)			
Severe motor delay	40	1	12	21	6	0.009	0.000	0.783
(1=yes)	(4.04)	(0.51)	(7.64)	(12.88)	(2.45)			
Any of severe delay	237	57	48	67	65	0.995	0.112	0.944
(1=yes)	(29.44)	(29.23)	(30.57)	(41.10)	(26.53)			
Any of two types of severe delay	84	20	18.00	26	20	0.987	0.382	0.917
(1=yes)	(10.44)	(10.26)	(11.46)	(15.95)	(8.16)			
Any of three types of severe delay	26	4	9	9	4	0.328	0.372	0.997
(1=yes)	(3.56)	(2.05)	(5.73)	(5.52)	(1.63)			
Four types of severe delay	5	0	2	3	0	0.468	0.144	1.000
(1=yes)	(0.56)	(0.00)	(1.27)	(1.84)	(0.00)			
Observations	760	195	157	163	245			

Values are presented as frequencies, with percentages in parentheses. Percentages are weighted using sampling weights

We also report the proportion of severe delays in Table 4.. The results show that the prevalences of severe delays among children in cognitive, language, social-emotional, and motor are 17%, 14%, 9%, and 4%, respectively. The proportion of children with any type of severe delay is 29%, while 10% have delays in at least two domains and 5% in three or more areas.

### Family care indicators across subpopulations

Table 5 shows the scores for the overall FCIs, the scores for each of the 5 subscales, and the proportion of families who use or engage in each item in the FCI subscales, both in the full sample and for each of the four subpopulation samples. In the full sample, we find that families have an average of 2.73 sources of play material out of a possible 4, and the average score for varieties of play materials is 4.91 out of a possible 7. On average, family members regularly engage with their child in 3 out of 6 play activities. When looking at the proportion of families who use or engage in each item in the five FCI subscales, we find that, overall, the families have relatively low scores on most FCI subscales and the individual items that comprise the subscales. Specifically, less than 70% of the families indicate that they use or practice half or more of the items. Only several items are used or practiced by a large share of the families; Examples of these items include “homemade toys” (97%), “things for moving around” (94%), and “taking the child outside the home” (85%). In addition, 56% of the families in our full sample have fewer than two adult books at home, and 67% do not have magazines or newspapers at home.

**Table 5:** Family care indicators

	Full sample	Mountainous Rural populations	Central rural populations	Resettlement populations	Migrant populations	Difference between populations		
	Mean	Mean	Mean	Mean	Mean	*p*-value		
	(SD)	(SD)	(SD)	(SD)	(SD)			
						(2)-(3)	(2)-(4)	(2)-(5)
	(1)	(2)	(3)	(4)	(5)	(6)	(7)	(8)
Total score (0–19)	11.77	11.88	11.41	10.06	12.44	0.687	0.000	0.426
	(3.57)	(3.76)	(3.43)	(3.96)	(3.34)			
Sources of play materials (0–4)	2.73	2.81	2.75	2.40	2.56	0.960	0.002	0.075
	(1.00)	(0.98)	(0.99)	(1.00)	(1.02)			
Household objects (1 = yes)	0.37	0.37	0.36	0.36	0.37	0.997	0.421	0.991
	(0.48)	(0.49)	(0.48)	(0.48)	(0.48)			
Things from outside (1 = yes)	0.74	0.73	0.71	0.76	0.74	0.899	0.989	0.991
	(0.44)	(0.45)	(0.45)	(0.43)	(0.44)			
Toys bought from store (1 = yes)	0.65	0.73	0.51	0.65	0.65	0.491	0.000	0.000
	(0.48)	(0.45)	(0.50)	(0.48)	(0.48)			
Homemade toys (1 = yes)	0.97	0.98	0.97	0.97	0.97	0.993	0.000	0.963
	(0.16)	(0.14)	(0.17)	(0.16)	(0.16)			
Variety of play materials (0–7)	4.91	5.00	4.65	4.04	5.39	0.294	0.000	0.129
	(1.72)	(1.71)	(1.74)	(1.97)	(1.52)			
Things that make/play music (1 = yes)	0.89	0.90	0.86	0.75	0.95	0.649	0.000	0.528
	(0.31)	(0.30)	(0.35)	(0.44)	(0.22)			
Things for drawing/writing (1 = yes)	0.53	0.57	0.48	0.36	0.57	0.414	0.001	1.000
	(0.50)	(0.50)	(0.50)	(0.48)	(0.50)			
Picture books for children (not school books) (1 = yes)	0.54	0.54	0.49	0.40	0.64	0.802	0.075	0.245
	(0.50)	(0.50)	(0.50)	(0.49)	(0.48)			
Things meant for stacking, constructing, building (blocks) (1 = yes)	0.66	0.64	0.62	0.53	0.80	0.976	0.202	0.006
	(0.47)	(0.48)	(0.49)	(0.50)	(0.40)			
Things for moving around (e.g., balls, bats) (1 = yes)	0.94	0.95	0.92	0.90	0.97	0.803	0.214	0.878
	(0.24)	(0.22)	(0.27)	(0.31)	(0.18)			
Toys for learning shapes and colors (1 = yes)	0.62	0.58	0.61	0.55	0.73	0.984	0.904	0.020
	(0.49)	(0.49)	(0.49)	(0.50)	(0.44)			
Things for pretending (e.g., dolls, tea set) (1 = yes)	0.73	0.81	0.67	0.55	0.73	0.040	0.000	0.376
	(0.44)	(0.40)	(0.47)	(0.50)	(0.44)			
Play activities in the past 3 days (0–6)	3.36	3.35	3.29	2.85	3.56	0.990	0.044	0.667
	(1.68)	(1.77)	(1.60)	(1.78)	(1.66)			
Read books or look at picture books with child (1 = yes)	0.31	0.32	0.30	0.24	0.33	0.973	0.41	0.999
	(0.46)	(0.47)	(0.46)	(0.43)	(0.47)			
Tell stories to child (1 = yes)	0.29	0.32	0.25	0.25	0.36	0.567	0.522	0.868
	(0.46)	(0.47)	(0.43)	(0.43)	(0.48)			
Sing songs with child (1 = yes)	0.52	0.50	0.50	0.46	0.61	1.000	0.920	0.149
	(0.50)	(0.50)	(0.50)	(0.50)	(0.49)			
Take child outside home (1 = yes)	0.85	0.82	0.90	0.77	0.84	0.186	0.638	0.857
	(0.35)	(0.39)	(0.30)	(0.42)	(0.36)			
Play with child with toys (1 = yes)	0.76	0.71	0.78	0.67	0.80	0.499	0.814	0.212
	(0.43)	(0.45)	(0.41)	(0.47)	(0.40)			
Spend time with child in naming things, counting, drawing (1 = yes)	0.62	0.69	0.56	0.47	0.62	0.114	0.000	0.507
	(0.49)	(0.46)	(0.50)	(0.50)	(0.49)			
Household books								
Over two adult books in household (1 = yes)	0.44	0.37	0.42	0.44	0.59	0.858	0.710	0.000
	(0.50)	(0.49)	(0.50)	(0.50)	(0.49)			
Magazines or newspapers in household								
Magazines and newspapers for adult (1 = yes)	0.33	0.34	0.30	0.34	0.36	0.895	1.000	0.987
	(0.47)	(0.47)	(0.46)	(0.47)	(0.48)			
Observations	760	195	157	163	245			

There also are differences among the subpopulations when examining the overall FCIs, the FCI subscales, and the individual items. As compared with mountainous populations, resettlement populations have significantly lower scores on total FCIs. The total FCI score (SD) of resettlement populations is 10.06 (3.96) compared to 11.88 (3.76) in mountainous populations. Resettlement populations also score significantly lower than do mountainous populations in terms of sources of play materials and variety of play materials. Resettlement populations score only 2.40 (1.0) in sources of play materials compared to 2.81 (0.98) among mountainous populations. In addition, resettlement populations score 4.04 (1.97) in the variety of play materials, while mountainous populations score 4.85 (1.79). Compared to the subpopulation that resides in the mountainous regions, we find that there are significantly lower shares of families in resettlement populations that have FCIs in terms of “things from outside,” “toys bought from a store,” “things that play music,” “things for drawing,” and “things for pretending.” A significantly greater share of families in migrant populations use “things meant for stacking, constructing, building” and “toys for learning shapes and colors.” We also find that a significantly greater proportion of families in migrant populations have more than two adult books at home (59%) compared to mountainous populations (37%). The results presented in Table 4. allow us to see the sources of the generally low levels of FCIs for overall China and for the different subpopulations.

### Factors associated with FCIs

Using the same model, Table 6 shows the associations between child and household characteristics and FCIs. When examining child characteristics, we find that the child’s age is significantly associated with total FCI score as well as subscale scores for sources of play materials, variety of play materials, and number of adult books. Specifically, a one-month increase in the child’s age corresponds to a 0.06-point increase in the household’s FCI score (*p* =.000). It is also associated with a 0.04-point increase in the variety of play materials by (*p* =.000) and a 0.01-point increase in play activities (*p* =.084). The child being born prematurely is associated with a 60% increase in the likelihood that the household will have more than two adult books.

**Table 6:** Association between child and household characteristic and family care indicators

Variable	Family care indicators index	Sources of play materials	Varieties of play materials	Play activities in the past three days	Over two adult books in household	Magazines and newspapers for adult
	(1)	(2)	(3)	(4)	(5)	(6)
Child characteristics						
Age (in months)	0.06	0.00	0.04	0.01	−0.01	−0.00
	(0.01)	(0.00)	(0.01)	(0.01)	(0.01)	(0.00)
	[0.000]	[0.262]	[0.000]	[0.084]	[0.048]	[0.902]
Male (1 = yes)	0.19	−0.03	0.01	0.18	0.12	0.09
	(0.21)	(0.08)	(0.10)	(0.15)	(0.07)	(0.09)
	[0.356]	[0.684]	[0.894]	[0.254]	[0.082]	[0.344]
Premature (1 = yes)	0.71	0.06	0.12	0.23	0.60	−0.02
	(0.83)	(0.19)	(0.31)	(0.41)	(0.29)	(0.15)
	[0.399]	[0.752]	[0.697]	[0.581]	[0.045]	[0.874]
Household characteristics						
Primary caregiver (1 = mother)	1.31	0.28	0.34	0.49	0.23	0.17
	(0.36)	(0.14)	(0.20)	(0.13)	(0.14)	(0.11)
	[0.001]	[0.051]	[0.101]	[0.001]	[0.104]	[0.126]
Maternal age (1 = above 25 years)	0.55	0.27	0.30	−0.11	0.12	0.23
	(0.38)	(0.15)	(0.18)	(0.18)	(0.14)	(0.08)
	[0.154]	[0.072]	[0.103]	[0.551]	[0.392]	[0.009]
Maternal education level (1 = 9 years or higher)	1.74	−0.06	0.72	0.64	0.78	0.48
	(0.29)	(0.09)	(0.13)	(0.13)	(0.13)	(0.13)
	[0.000]	[0.466]	[0.000]	[0.000]	[0.000]	[0.000]
Household receives MLSG (1 = yes)	0.57	−0.02	0.20	0.15	0.27	0.42
	(0.51)	(0.20)	(0.16)	(0.29)	(0.16)	(0.20)
	[0.271]	[0.912]	[0.222]	[0.601]	[0.090]	[0.043]
Household asset index	0.49	−0.02	0.24	0.20	0.07	0.16
	(0.16)	(0.04)	(0.08)	(0.06)	(0.07)	(0.06)
	[0.004]	[0.614]	[0.003]	[0.001]	[0.327]	[0.006]
Observations	760	760	760	760	760	760

For each variable, the three rows represent the regression coefficient, standard error (in parentheses), and exact p-value, respectively. We do not display the coefficient of parental occupation. All models control for county fixed effects and population type fixed effects. Standard errors in parentheses are clustered at the village level to account for intra-village correlation. P-values are in brackets

*MLSG* Minimum Living Standard Guarantee

Our analysis of household characteristics finds that FCIs are significantly associated with whether the primary caregiver is the mother, maternal education level, and family asset level. Compared to families where the primary caregiver is not the mother, families where the primary caregiver is the mother scored 1.31 points higher in the total FCI score, and 0.49 points higher in play activities, respectively (all *p* =.001). In addition, families where the mother completed 12 or more years of education scored 1.74 points higher in the total FCI score, 0.72 points higher in variety of play materials, and 0.64 points higher in the number of play activities (all *p* =.000). Higher maternal education also is associated with a 78% greater likelihood of having more than two adult books in the household and a 48% greater likelihood of having magazines or newspapers in the household (both *p* =.000).

Finally, the household asset index is significantly associated with total FCIs, sources of play materials, variety of play materials, play activities, and magazines and newspapers for adults. Specifically, a one-point increase in household asset index is associated with an increase of 0.49 points in the total FCI score (*p* =.004). It is also associated with an increase of 0.24 points in the variety of play materials (*p* =.003), an increase of 0.20 points in the number play activities (*p* =.00), and a 16% higher likelihood of having magazines or newspapers for adults (*p* =.006).

### Family care indicators and early childhood development

Table 7 presents the associations between FCIs (total FCI score and the score for each of the five subscales) and ECD outcomes for the full sample. We find that the total FCI score is positively and significantly associated with all four of the developmental outcomes measured. A one-point increase in total FCI score correlates to a 0.05 SD increase in cognition (*p* =.001), a 0.05 SD increase in language development (*p* =.000), a 0.07 SD increase in social-emotional development (*p* =.000), and a 0.06 SD increase in motor development (*p* < = 0.013).

**Table 7:** Association between family care indicators and child development scores

Variable	Cognitive Scores	Language Scores	Social-emotional Scores	Motor Scores
	(1)	(2)	(3)	(4)
Family care index (scores)	0.05	0.05	0.07	0.06
	(0.01)	(0.01)	(0.01)	(0.02)
	[0.001]	[0.000]	[0.000]	[0.013]
Sources of play materials (scores)	−0.02	0.01	0.09	0.06
	(0.03)	(0.03)	(0.04)	(0.03)
	[0.444]	[0.831]	[0.029]	[0.101]
Varieties of play materials (scores)	0.11	0.07	0.13	0.07
	(0.03)	(0.02)	(0.03)	(0.04)
	[0.001]	[0.002]	[0.000]	[0.087]
Play activities (scores)	0.08	0.10	0.11	0.14
	(0.03)	(0.02)	(0.03)	(0.04)
	[0.002]	[0.000]	[0.000]	[0.004]
Over 2 adult books in household (1 = yes)	0.32	0.28	0.24	0.15
	(0.08)	(0.06)	(0.11)	(0.11)
	[0.000]	[0.000]	[0.041]	[0.181]
Magazines and newspapers for adult (1 = yes)	0.05	0.01	0.16	0.00
	(0.08)	(0.07)	(0.10)	(0.10)
	[0.534]	[0.839]	[0.127]	[0.964]
Observations	760	760	760	760

Each cell reports the result of a separate regression. For each variable, the three rows represent the regression coefficient, standard error (in parentheses), and exact p-value, respectively. All developmental scores are non-parametrically standardized by child age (in months). Control variables include child’s age, gender, premature birth status, maternal age, maternal education, whether the mother is the primary caregiver, whether the household receives welfare benefits, and a household asset index. The asset index is constructed using polychoric principal component analysis based on the presence of the following items: tap water, toilet, water heater, washing machine, computer, internet access, refrigerator, air conditioner, motor or electric bicycle, and car. All models include fixed effects for Bayley tester, county, and population type. Standard errors in parentheses are clustered at the village level. P-values are in brackets

Of the individual dimensions of FCIs, we find that the variety of play materials and play activities are positively associated with all four developmental outcomes. A one-point increase in the variety of play materials is associated with 0.11 SD (*p* =.001), 0.07 SD (*p* =.002), and 0.13 SD (*p* =.000) and a 0.07 SD (*p* =.087) increases in cognitive, language, and social-emotional development, respectively. Regarding play activities, a one-point increase corresponds to an improvement in cognitive development by 0.08 SD (*p* =.002), an improvement in language development by 0.10 SD (*p* =.000), an improvement in social-emotional development by 0.11 SD (*p* =.000), and an improvement in motor development by 0.14 SD (*p* =.004). In addition, the sources of play materials are significantly associated with social-emotional development but no other developmental outcomes. Specifically, a one-point increase corresponds to an improvement in social-emotional development by 0.09 SD.

We also find that parental investment in books is associated with better language and social-emotional development. Children in households with more than two adult books score higher in cognitive development by 0.32 SD (*p* =.000), language development by 0.28 SD (*p* =.000) and in social-emotional development by 0.24 SD (*p* =.041)compared to children in households with two or fewer than two adult books. The relationship between the number of books and other developmental outcomes, however, was not statistically significant. The number of magazines or newspapers in the home also was not significantly associated with any of the developmental outcomes measured.

## Discussion

We find that children in our sample have overall low developmental scores across the four rural subpopulations. Compared to a healthy population, the mean scores of each of four domains are lower. However, these findings are consistent with previous studies in rural China [20, 40], which have shown that the developmental scores of rural young children are relatively low.

Our results show that, across the four rural subpopulations, there is a high prevalence of developmental delays among children. In the case of cognitive, language, and social-emotional development, the rates of delay in our sample are three or more times higher than in a healthy population. Importantly, however, these findings are consistent with those of other studies of rural children across China [20, 40], indicating that the problem of developmental delays among rural children is widespread.

When we compare rates of delay between the four subpopulations, we discover that migrant populations have significantly lower rates of developmental delays than mountainous subpopulations. This finding is similar to the results of a study by Wang et al. [26], who found that migrant populations tend to have lower rates of developmental delays compared to other rural subpopulations. This is, in part, due to the higher socioeconomic status and education level of migrant populations [41]. The results are consistent with previous ECD studies which show that a child develops better if the household has high socioeconomic status and their parents have higher education levels [42, 43]. However, we note that, except for motor delays, the rates of developmental delay in our migrant subsample are much higher than those found in a healthy population.

When we compare FCI scores across subpopulations within our sample, we find that migrant populations have higher total FCIs and subscale scores than mountainous subpopulations. Meanwhile, although the variety of play materials among migrant households in our sample is comparable to that found among families in Malawi (a subscale score of 5.27) [15], it is much lower than those found in other developing countries [12].

In terms of correlates of FCIs, we find that the child’s age is significantly associated with total FCI score, sources of play materials, variety of play materials, and number of adult books. The strengths of these associations, however, are weak. While other studies of FCIs have not found a significant association between children’s age and FCIs scores, they reported stronger associations between FCIs and other characteristics [12]. We also find that the mother as the primary caregiver, maternal education level, and family asset level are significantly associated with higher total FCIs and subscale scores. These results are consistent with studies of parenting behaviors in rural China as well as studies of FCIs in other developing countries [12, 19–21]. These associations between household characteristics and FCIs may explain why migrant families have higher FCIs compared to mountainous rural families, central plains families, and resettlement families in our sample. Migrant families in our sample tend to have more educated mothers as well as higher family asset values, which may contribute to better FCI scores. Although FCI scores among migrant families are better than those of the other subpopulations in this study, it is important to note that migrant families still have lower FCIs than do samples in other developing countries [9, 15]. This means that, although migrant populations are doing relatively better than other rural subpopulations of China, there is still a need to improve FCIs among all rural subpopulations.

Finally, when we examine the associations between FCIs and ECD outcomes, we find that family care is significantly associated with ECD. Among our sample, increases in the variety of play materials and play activities were significantly associated with better outcomes in terms of cognitive, language, social-emotional, and motor development. These results are consistent with those of numerous studies in other countries, which have found positive associations between the variety of play materials, play activities, and ECD outcomes [15, 16]. Our finding that play activities are significantly associated with developmental outcomes is also consistent with those of studies in rural China that have linked interactive parenting to better developmental outcomes [19–21]. The relatively low variety of play materials and play activities we observed may explain the high rates of developmental delays among the sample children.

By investigating developmental outcomes in four major rural subpopulations across different regions of China, this study sheds light on a serious issue across rural China. The results suggest that the high rates of developmental delays among toddlers are due partly to poor family care indicators. Despite the suboptimal FCIs and prevalent ECD delays across rural China, the literature has not addressed this issue. The results of this study have implications for ECD policies and programs in rural China. Based on the high rates of developmental delays across the four subpopulations in our sample, we call for policymakers to increase their attention to improving the developmental outcomes of children aged 0–3 years in China’s rural areas. This study provides evidence that improving the quality of parenting and, more specifically, the variety of play materials and interactions between caregivers and children may be key to improving the developmental outcomes of rural children. We recommend that policymakers develop parenting intervention programs to improve the parenting practices in rural subpopulations across China. The results of our study also suggest that certain groups should be specifically targeted for such programs, notably poorer families, families in which the mother is not present or is not the primary caregiver, and those in which caregivers have lower education levels.

This study makes two major contributions to the literature. First, unlike other studies in developing settings that have examined only a few aspects of the family care environment and studies in rural China that have measured only interactive parenting practices, we examine multiple aspects of parenting quality using the FCIs survey. Second, our study is the first study to compare parenting quality and analyze the associations between parenting quality and ECD across different subpopulations within a large developing setting.

We also acknowledge several limitations of this study. First, this study relies on cross-sectional, non-experimental data, which does not allow us to identify causal relationships. Future research should examine causal connections between FCIs and ECD to confirm the results of this study. Second, the data we collected on the FCIs and the household asset index relied on self-reporting by caregivers, which cannot rule out the possibility of recall and self-reporting bias. Third, even though we tried to include the most relevant confounders in our analysis (that is, the variables that measure the sample child and household characteristics in the regression analysis), some confounders that could affect either child development or parenting quality might be omitted. For example, the caregiver’s level of depression, anxiety, and stress can affect parenting practices, which in turn can have an effect on child development. In addition, caregivers with mental health problems may negatively impact child development through a stressful home environment. Unfortunately, we did not collect such data in this study. Future studies should include caregiver mental health and other similar types of covariates. Fourth, although our dataset covers four major rural subpopulations in China, our sample does not include rural households in eastern China. Considering this, we do not claim that they are fully representative of these areas. Future research should examine parenting quality across all rural subpopulations to achieve a more generalizable understanding of parenting quality in China’s rural communities.

## Supplementary Information

Supplementary Material 1

## Data Availability

The data that support the findings of this study are available from the corresponding author, upon reasonable request.

## Abbreviations

ECD: Early childhood development
FCIs: Family Care Indicators
Bayley-III: Third Edition of the Bayley Scales of Infant and Toddler Development
UNICEF: United Nations Children’s Fund
