# Supplementary material for: Parenting quality and early childhood development: evidence from different rural subpopulations in China
Source: BMC Psychol. 2025 Nov 18;13:1273. doi: 10.1186/s40359-025-03580-5 (PMC12625244; doi:10.1186/s40359-025-03580-5)
Supplement: Supplementary file 1 — Supplementary Material 1 [file 40359_2025_3580_MOESM1_ESM.docx]

### Supplementary File

### The distribution of four rural subpopulations

In China, nearly 70% of the nation’s children are from some type of rural population: mountainous rural, plains rural, migrant, and resettlement (National Bureau of Statistics of the People’s Republic of China, 2010). Based on the 2015 Micro-Census, Wang et al. (2019) estimated that there are 12 million children who are growing up among mountainous populations; 13 million, among plains rural populations; 600,000, among resettlement populations; and 6 million, among migrant populations.

Mountainous populations are located chiefly in 12 western provinces (Xinjiang, Tibet, Qinghai, Ningxia, Yunnan, Sichuan, Guangxi, Guizhou, Gansu, Shaanxi, Inner Mongolia, and Chongqing) as well as within prefectures and the counties of Hebei, Henan, Anhui, and Jiangxi Provinces, which have a lower economic status. The annual per capita disposable income of these rural areas was 10,828 RMB ($1,370 USD) in 2017. Plains rural populations can be found in seven central provinces (Henan, Hubei, Hunan, Anhui, Jiangxi, Jilin, and Heilongjiang) as well as within prefectures and the counties of Shaanxi, Hebei, and Shandong Provinces, which have a bit higher economic status than do western rural areas. The annual per capita disposable income of these rural areas was 12,805 RMB ($1,620 USD) in 2017 (National Bureau of Statistics of the People’s Republic of China, 2018).

China has nationally designated poverty counties that have been established by the central government. The population of these nationally designated counties has a large share that is low-income and needs higher levels of support from the central government. Most of China’s nationally designated poverty counties are located in mountainous areas. The threshold for being designated a poverty county (when they were established in the late 1990s and early 2000s) was that the annual per capita income of a county’s resident was less than 2,300 RMB, or about $1 USD per person per day (The State Council Leading Group Office of Poverty Alleviation and Development, 2012). In 2017, there were 572 nationally designated poverty counties across China.

Anti-poverty initiatives have moved 12 million rural individuals from mountainous areas into resettlement communities, starting from the 11th Five-Year Plan in 2006 and continuing to the present (National Development and Reform Commission, 2006, 2012, 2016). Resettlement communities are designed to be in locations that offer convenient transportation to county seats and urban areas. Communities also are supposed to be located in areas that are less vulnerable to natural disasters than the original communities in more remote rural areas. Resettlement populations are concentrated in dense, apartment-like living arrangements. In general, however, they still have a lower economic status, as the families have just recently relocated from remote mountainous areas. The annual per capita disposable income of these areas was 9,377 RMB ($1,187 USD) in 2017 (National Bureau of Statistics of the People’s Republic of China, 2018).

As China has urbanized over the past several decades, a large flow of rural migrants has come to urban areas in search of jobs. Many migrant communities have formed inside the boundaries of urban districts (Huang, 1999). Starting in the 1990s, nearly 250 million individual migrants have moved into first-, second-, or third-tier cities. First-tier cities are large metropolitan areas that play an important role in the economy and politics of the country. Second-tier cities are slightly smaller but economically prosperous cities with political importance. Third-tier cities are smaller and less powerful than are second-tier cities (National Bureau of Statistics of the People’s Republic of China, 2020). These migrant populations are originally from rural areas across China. In general, rural migrants have a higher economic status and, in many cases, higher levels of education or in some way have obtained special job training compared to those in other rural subpopulations (Liu, 2004).

**Assessment of regression model assumptions**

To ensure the validity and reliability of the regression models, we assessed the assumptions of the regression models: linearity, normality of residuals, homoscedasticity, independence of errors, and multicollinearity.

**1. Linearity**

Scatter plots were used to visually assess the linearity assumption. In our study, the independent variables are indicators of FCI, while the dependent variables are child developmental outcomes. These plots (Figure 1 in below) do not reveal any strong non-linear patterns, suggesting that the relationship between indicators of FCI and child development is approximately linear.

Figure 1. Linear relationship between FCI scores and child development

**2. Normality of residuals**

This assumption requires that residuals (i.e., the differences between the observed and predicted values) are normally distributed. We used histogram to visually assess this assumption (Figure 2). The histograms suggest that the residuals are approximately normally distributed. In addition, the Shapiro –Wilk test was applied to formally assess normality. Although the Shapiro –Wilk test was significant for the language scores model, likely due to the large sample size, visual inspection of the histogram indicated no substantial deviation from normality.

a. Cognition b. Language

c. Social-emotion d. Motor

Figure 2. Normality of residuals

**3. Homoscedasticity**

We plotted the standardized residuals against the predicted values to check for homoscedasticity. The points were randomly dispersed around zero without forming obvious funnel or patterns (Figure 3), thus satisfying the homoscedasticity assumption. We also conducted Breusch-Pagan tests, which were non-significant (p > 0.05) for all models, confirming constant variance.

a. Cognition b. Language

c. Social-emotion d. Motor

Figure 3. Homoscedasticity of errors

**4. Independence of errors**

The Durbin-Watson statistic was calculated for each model. The values were all close to 2 (ranging from 2.03 to 2.13), indicating no serious autocorrelation in the residuals and supporting the assumption of independence.

**5. Multicollinearity**

We assessed multicollinearity by calculating the Variance Inflation Factor (VIF). All VIF values were below 2, well under the common threshold of 10, indicating that multicollinearity was not a concern in our models.

All tested assumptions of linear regression were satisfied. The results confirm that the models in our study are appropriate for interpretation, and the parameter estimates are valid and reliable.

**References**

Huang, C. (1999). Characteristics of migrant population and community management in large cities—A case study of Shanghai. *Population Studies*. http://www.cqvip.com/QK/95654X/199904/1002158727.html

Liu, J. (2004). *Employment and transfer of rural labor force*. http://www.china.com.cn/chinese/zhuanti/0304jybg/741115.htm

National Bureau of Statistics of the People’s Republic of China. (2010). *China Statistical Yearbook 2010*. <http://www.stats.gov.cn/tjsj/ndsj/2010/indexeh.htm>

National Bureau of Statistics of the People’s Republic of China. (2018). *China Statistical Yearbook 2018*. <http://www.stats.gov.cn/tjsj/ndsj/2018/indexeh.htm>

National Bureau of Statistics of the People’s Republic of China. (2020). *Statistical bulletin of the People’s Republic of China on national economic and social development in 2019*. http://www.stats.gov.cn/tjsj/zxfb/202002/t20200228_1728913.html

National Development and Reform Commission. (2006). *China’s 11th Five Year Plan for poverty alleviation and relocation.* https://www.ndrc.gov.cn/fzggw/jgsj/dqs/sjdt/200612/t20061227_1091414.html

National Development and Reform Commission. (2012). *China’s 12th Five Year Plan for poverty alleviation and relocation.* https://www.ndrc.gov.cn/fzggw/jgsj/dqs/sjdt/201208/t20120813_1058864.html

National Development and Reform Commission. (2016). *China’s 13th Five Year Plan for poverty alleviation and relocation.* https://www.ndrc.gov.cn/xxgk/zcfb/ghwb/201610/t20161031_962201.html

The State Council Leading Group Office of Poverty Alleviation and Development. (2012). *List of counties in contiguous poverty-stricken areas in China*. http://www.gov.cn/gzdt/2012-06/14/content_2161045.htm

Wang, L., Liang, W., Zhang, S., Jonsson, L., Li, M., Yu, C., Sun, Y., Ma, Q., Bai, Y., Abbey, C., Luo, R., Yue, A., & Rozelle, S. (2019). Are infant/toddler developmental delays a problem across rural China? *Journal of Comparative Economics*. https://doi.org/10.1016/j.jce.2019.02.003
